# Interaction Between Smoking and Internet Gaming Disorder on Spontaneous Brain Activity

**DOI:** 10.3389/fpsyt.2020.586114

**Published:** 2020-12-03

**Authors:** Xianxin Qiu, Xu Han, Yao Wang, Weina Ding, Yawen Sun, Yan Zhou, Hao Lei, Fuchun Lin

**Affiliations:** ^1^National Center for Magnetic Resonance in Wuhan, State Key Laboratory of Magnetic Resonance and Atomic and Molecular Physics, Wuhan Institute of Physics and Mathematics, Innovation Academy for Precision Measurement Science and Technology, Chinese Academy of Sciences, Wuhan, China; ^2^University of Chinese Academy of Sciences, Beijing, China; ^3^Department of Radiology, School of Medicine, Renji Hospital, Shanghai Jiaotong University, Shanghai, China

**Keywords:** interaction, internet gaming disorder, smoking, spontaneous brain activity, ALFF

## Abstract

Converging lines of evidence indicates that smoking and internet gaming disorder (IGD) affect spontaneous brain activity, respectively. However, little is known about whether these two factors work together on the human brain. In this study, we investigated the interaction between smoking and IGD on local spontaneous brain activity using amplitude of low-frequency fluctuation (ALFF) based on resting-state fMRI (rs-fMRI). Forty-six cigarette smokers, 38 IGD individuals, 34 participants with both IGD and cigarette smoking (IGD-Smoking), and 60 healthy individuals involved in the study. Voxel-wise analysis of covariance of ALFF revealed that there were significant interactions between IGD by smoking in the right medial pre-frontal cortex (MPFC)/ventral striatum, bilateral cerebellar, and visual-related regions as well as the left temporal gyrus. In the right MPFC/ventral striatum and left temporal gyrus, ALFF in smoking group was significantly higher than healthy group while there were no significant ALFF differences between IGD-Smoking group and IGD group. While in the bilateral cerebellar and visual-related regions, ALFF in the smoking group was significantly lower than healthy group while ALFF in IGD-Smoking group did not show significant difference with IGD group. In addition, in the smoking group, ALFF of the right MPFC/ventral striatum was associated positively with anxiety and depression scores while the ALFF value in the smoking group had a trend toward negative correlation with SDS scores in the bilateral cerebellar and visual-related regions. The ALFF value in the smoking group was associated positively with anxiety score in the left temporal gyrus. These findings indicate that smoking and IGD interacted with each other in the human brain. Our results, in terms of spontaneous brain activity, may imply the fact that IGD people are more tended to get smoking. Moreover, it is possible to predict that smokers may be more easily to get internet addiction than healthy people.

## Introduction

Addiction is a complex phenomenon involving psychological and social consequences such as dependence, tolerance, sensitization, and craving (1, 2). Internet gaming disorder (IGD) is classified as an addictive disorder in the Diagnostic and Statistical Manual of Mental Disorders (5th Edition, DSM-5), characterized by the use of online games in a manner that leads to significant distress and functional impairments of general life (3). Smoking is one of the largest single causes of preventable morbidity and natural mortality, which may also cause cognitive decline and dementia (4). IGD and smoking, the typical representative of behavioral and substance addiction, respectively, have common core clinical features: diminished control and a hedonic quality over the problematic behavior, unsuccessful trying to stop, and impairment of major life functioning (5), and may share similar neural circuits including the reward circuits, memory and learning circuits, cognitive control loops (6, 7).

Studies based on questionnaires suggest that the youthful initiation of internet addiction in teenage students could be predicted by smoking (8–10). Subjects with both nicotine dependence and IGD demonstrated a greater degree of urge activities in the anterior cingulate cortex (ACC) and parahippocampus in comparison to the controls (11). Another study showed that compared with the non-smokers with IGD, smokers with IGD had motivation and executive function changes (12). Additionally, compared with healthy controls, both IGD and smoking groups showed significantly different resting-state functional connectivity (rsFC). Significant rsFC differences were also found between IGD and smoking groups (13).

Resting-state fMRI (rs-fMRI) has been shown to study spontaneous brain activity by assessing the baseline brain activity based on the low-frequency (<0.1 Hz) fluctuations of blood oxygenation level-dependent signals. Amplitude of low-frequency fluctuation (ALFF) has been applied to detect abnormalities of brain activity in IGD individuals and smokers, respectively, by measuring the local spontaneous brain activity (14). Compared with healthy controls, IGD individuals had increased ALFF in brain areas of the bilateral middle cingulate cortex, right parahippocampal gyrus, left precuneus, supplementary motor area, and medial orbitofrontal cortex (OFC) (15). Abnormal OFC and ACC activation might be associated with cue-induced gaming urge (16). The OFC connects extensively with the striatum and limbic regions and it is involved in cognitive and impulse control and reward processing by assessing the motivational significance of stimuli and selecting behavior to achieve desired outcomes (15). So abnormal OFC activity in addiction might be related to dysfunction of cognitive control ability (15). Activation was also found in ACC in IGD group when compared to the control group in resting state (17). ACC involved in modulating emotion, motivation and attention and monitoring conflicts to trigger desired execution and control outputs (18). Increased fractional ALFF (fALFF) was found in the superior temporal gyrus. The temporal gyrus serves as regulating sense perception including visual and auditory and its nerve fibers project to frontal lobe (19). Compared to non-smokers, smokers showed decreased fALFF in the precuneus and cerebellum anterior lobe (20, 21). Diminished regional homogeneity of bilateral cuneus and lingual gyrus was found in heroin-dependent individuals (22) and regional cerebral blood flow of bilateral cuneus was also decreased in opioid addiction (23). The major function of bilateral cuneus is visual processing and inhibitory control. This abnormity may suggest the impaired inhibitory control ability in addicted subjects. All these reports have studied the effects of IGD or smoking on ALFF in brain areas, respectively. However, less is known about the changes on spontaneous brain activity when subjects have both IGD and smoking disorders and whether they work on human brain independently or interactively in alterations of brain functions.

The primary goals of this study were to explore the effects of smoking and IGD on spontaneous brain activity. Given the high IGD risk associated with smoking and high smoking risk associated with IGD mentioned in many studies (9, 24), we hypothesized that IGD and smoking were not independently working on human brain. Therefore, in this study we investigate the interaction between smoking and IGD on the spontaneous brain activity. Four groups of healthy controls, smokers, IGD and IGD-Smoking individuals were under resting-state fMRI scanning. ALFF was calculated and the voxel-wise two-way analysis of covariance (ANCOVA) was performed to investigate the interaction between smoking and IGD.

## Materials and Methods

### Subjects

One hundred and eighty-five right-handed subjects, including 62 healthy controls (age range: 16–28 years), 50 cigarette smokers (age range: 18–29 years), 39 IGD addicts (age range: 16–27 years), and 34 participants with both IGD and cigarette smoking (IGD-Smoking) (age range: 16–28 years) were involved in this study. Psychiatric medical disorders were screened by the Mini International Neuropsychiatric Interview for (25). All subjects did not have any drug abuse or dependence, psychiatric or neurological diseases history (other than nicotine and internet dependence for smokers and IGD individuals, respectively), or intellectual disability. All participants were cleared of substance addiction by a urine drug screening. Smokers were defined as those who smoked at least 10 cigarettes a day during the last year. Fagerström test for nicotine dependence (FTND) was used to evaluate the severity of smoking addiction (26). None of the smokers had been abstinent for more than 3 months in the previous year. Non-smokers smoked no more than five cigarettes during their lifetime. The IGD participants were enrolled from the psychological outpatient clinic at the Shanghai Mental Health Center and were interviewed by two experienced psychiatrists with the criteria of modified Young's Diagnostic Questionnaire for Internet Addiction (YDQ) (27). Ko et al. Internet Addiction Scale (CIAS) was performed to access the severity of internet addiction (28). Before MRI scanning, all participants were assessed by questionnaires of the Self-rating Anxiety Scale (SAS) (29), Self-rating Depression Scale (SDS) (30) and Barratt Impulsiveness Scale (BIS) (31).

### Image Acquisition

All subjects were scanned by a 3.0-Tesla GE Signa HDx (Milwaukee, WI, United States) with a standard 8-channel head coil and the head motion and scanner noise were minimized by foam pads. The parameters of the echo-planar imaging sequence for the rs-fMRI data collection were as follows: repetition time/echo time: 2,000/24 ms; matrix: 64 × 64; flip angle: 90°; field of view: 230 × 230 mm^2^; 4 mm with no gap; 34 slices. The rs-fMRI scanning lasted for 440 s and 220 volumes were obtained. All participants were instructed to lie still, keep their eyes closed but not to fall asleep during MRI scanning. All the resulted images were visually accessed by two experienced neuroradiologists to exclude pathological findings.

### Data Pre-Processing

Images were pre-processed by the Data Processing Assistant for Resting-State fMRI (32). The first 10 time points of each subject were excluded and the remaining 210 volumes were corrected for the acquisition time delay. Participants with a maximum head motion >2 mm in the x, y, or z direction or a head rotation >2° or mean framewise displacement larger than 0.2 mm were excluded. Using this criterion, 2 healthy controls, 4 smokers and 1 IGD addict were discarded, and a total of one hundred and seventy-eight subjects (60 healthy controls, 46 cigarette smokers, 38 IGD addicts, and 34 IGD-Smoking participants) were ultimately used in the ALFF analysis. The regression of head motion effects was carried out by Friston 24-parameter model (33). After that, the images normalization to the Montreal Neurological Institute (MNI) space and resampling to a 3-mm isotropic voxel and the smoothing procedure with a 6 mm full width at half maximum Gaussian kernel to reduce noises was performed. Linear and quadratic trends as well as white matter and corticospinal fluid signals were then removed. Finally, individual ALFF map was calculated within a mask without non-brain tissue, divided by its mean ALFF for further statistical analysis.

### Statistical Analysis

The voxel-wise two-way (IGD and smoking) ANCOVA controlling for age, gender, years of education and mean frame-wise displacement was performed to investigate the main effects as well as the interaction between smoking and IGD on ALFF. The multiple comparisons correction of statistical F-maps was performed with family-wise error (FWE) cluster-corrected (*p* < 0.05) when using a primary voxel determining threshold of *p* < 0.001 to protect against false-positive findings. Then ALFF values from significant clusters showing interaction effects were extracted to perform the *post-hoc* pairwise comparison controlling for age, gender, educational level and head motion and a Bonferroni correction level of 0.0083 (0.05/6) was applied to protect against false-positive findings. The correlation analysis with ALFF among groups as dependent variable and variables related to smoking such as duration of smoking, age at first smoking, and FTND and IGD related variables (CIAS), questionnaire scores of SAS, SDS, BIS and its three dimensions controlling for age, gender, educational level, and head motion was performed to investigate whether the altered ALFF was related to the smoking and IGD related features and questionnaire scores. Results with *p* < 0.05 (uncorrected) were considered significant.

## Results

### Demographic and Clinical Measures

The four groups showed significant sex differences according to the Chi-square test (*p* = 0.008). Other demographic and clinical differences of subjects among groups was evaluated with a two-way ANOVA in age, education level, CIAS, SAS, SDS, and BIS. There was significant main effect of IGD (*p* = 0.006) and smoking (*p* < 0.001) and IGD by smoking interaction (*p* = 0.004) on age. Significant main effect of IGD (*p* < 0.001) and smoking (*p* = 0.003) and no significant IGD by smoking interaction (*p* = 0.06) were also found in years of education (Table 1).

**Table 1:** Demographic and clinical characteristics of four groups.

	**IGD group (*n* = 38)**	**IGD-smoking group (*n* = 34)**	**HC group (*n* = 60)**	**Smoking group (*n* = 46)**		***p*-value**	
					**IGD**	**Smoking**	IGD* smoking
Age	19.66 ± 2.60	22.94 ± 2.93	22.29 ± 3.48	22.88 ± 2.78	0.006	<0.001	0.004
Gender (M/F)	26//12	33//1	41//19	31//15	–	–	0.008^a^
Education	10.74 ± 1.75	10.29 ± 2.10	14.76 ± 3.39	11.83 ± 2.50	<0.001	0.003	0.06
Duration of smoking	–	3.75 ± 1.88	–	5.18 ± 2.92	–	–	–
Age at first smoking	–	19.19 ± 2.83	–	17.70 ± 3.01	–	–	–
FTND	–	6.68 ± 2.07	–	6.28 ± 2.25	–	–	–
CIAS score	72.68 ± 10.27	81.26 ± 10.69	43.83 ± 10.83	49.93 ± 10.57	<0.001	<0.001	0.45
SAS score	48.89 ± 9.57	57.24 ± 11.56	40.53 ± 7.40	47.59 ± 9.93	<0.001	<0.001	0.66
SDS score	52.08 ± 9.41	58.00 ± 8.90	44.32 ± 8.62	50.65 ± 9.70	<0.001	<0.001	0.88
BIS-11 score	61.63 ± 8.19	64.12 ± 8.62	52.80 ± 6.91	55.67 ± 10.00	<0.001	0.039	0.88
BIS-attentional impulsiveness score	15.16 ± 2.69	15.97 ± 2.90	12.98 ± 2.30	13.13 ± 2.80	<0.001	0.24	0.41
BIS-motor impulsiveness score	20.63 ± 4.46	21.82 ± 3.64	17.92 ± 2.96	19.91 ± 4.00	<0.001	0.006	0.48
BIS-Non-planning impulsiveness score	26.16 ± 3.48	26.50 ± 4.66	21.90 ± 4.47	22.63 ± 5.62	<0.001	0.45	0.79

a *The Chi-square test showed significant sex differences within the four groups (p = 0.008)*.

*Values are expressed as mean ± standard deviation. The age, educational level, age at first cigarette, and duration of smoking are displayed in years. FTND, Fagerström Test of Nicotine Dependence; CIAS, Chen internet addiction scale; SAS, Self-rating Anxiety Scale; SDS, Self-rating Depression Scale; BIS-11, Barratt Impulsiveness Scale, version 11. The definition of educational level was the number of years of scholarship since primary school*.

Duration of smoking and age at first smoking in smoking group was longer (*p* = 0.01, *p* = 0.03, respectively), than that in IGD-Smoking individuals. IGD-Smoking group had higher FTND score than smoking group (*p* = 0.04). The main effect of IGD on CIAS (*p* < 0.001), SAS (*p* < 0.001), SDS (*p* < 0.001), BIS (*p* < 0.001) was significant. CIAS (*p* < 0.001), SAS (*p* < 0.001), SDS (*p* < 0.001), BIS (*p* = 0.039) in smokers were lower than non-smokers.

### Interaction Effects Between IGD and Smoking

**Figure 1:**
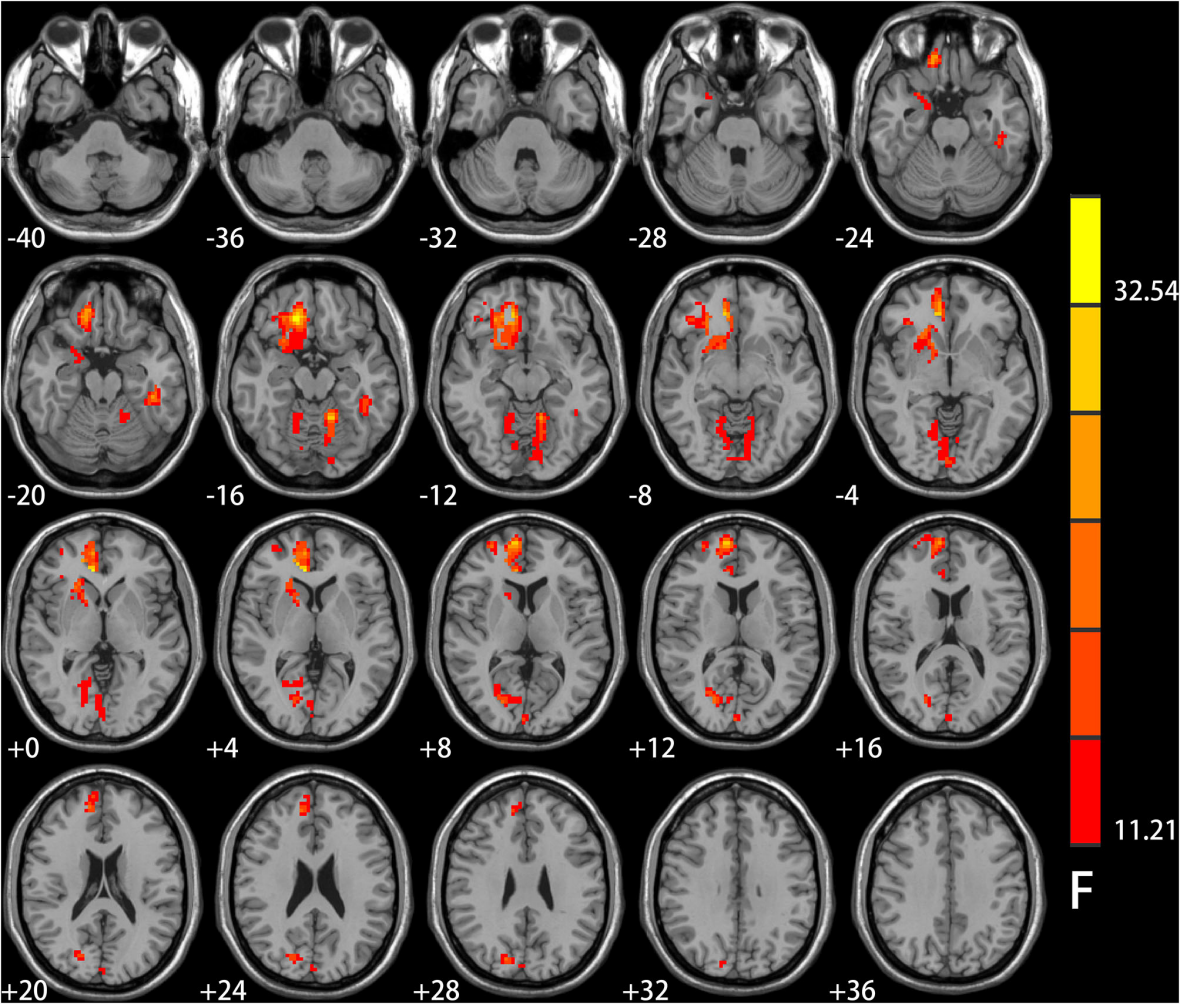
Results of ANCOVA analysis controlling for age, gender, educational level, and head motion. Brain regions showed group differences among the four groups of healthy controls, smokers, internet gaming disorder (IGD), and IGD-Smoking individuals in amplitude of low-frequency fluctuation (ALFF) (*p* < 0.05, FWE-corrected). The brain regions mainly involved in the right medial pre-frontal cortex (MPFC, i.e., orbital frontal gyrus and anterior cingulate cortex) extending to ventral striatum, bilateral cerebellar, and visual-related regions (i.e., lingual and calcarine gyrus and cuneus) as well as the left temporal gyrus.

Significant IGD and smoking interaction effects on ALFF were identified in the right medial pre-frontal cortex (MPFC) (i.e., orbital frontal gyrus and anterior cingulate cortex) extending to the ventral striatum, bilateral cerebellar and visual-related regions (i.e., lingual and calcarine gyrus and cuneus) as well as the left temporal gyrus (Figure 1 and Table 2).

**Table 2:** Regions showing significant amplitude of low-frequency fluctuation (ALFF) differences among the four groups of healthy control, smokers, internet gaming disorder (IGD), IGD-Smoking individuals (*p* < 0.05, FWE-corrected).

**Identified brain regions**	**Peak coordinates (MNI)**			**Side**	**Peak F**	**Cluster size (voxels)**
	**X**	**Y**	**Z**			
Medial pre-frontal cortex (i.e., orbital frontal cortex and anterior cingulate cortex)/ventral striatum	15	33	−15	R	36.81	863
Cerebellar and visual-related regions (i.e., calcarine, cuneus, and lingual gyrus)	−12	−51	−15	B	28.39	502
Inferior temporal gyrus	−42	−33	−21	L	25.28	71

*Analysis of covariance (ANCOVA) controlling for age, years of education, and mean frame-wise displacement was performed to investigate the interaction between smoking and IGD on ALFF*.

### Effect of IGD on Healthy and Smoking Groups

**Figure 2:**
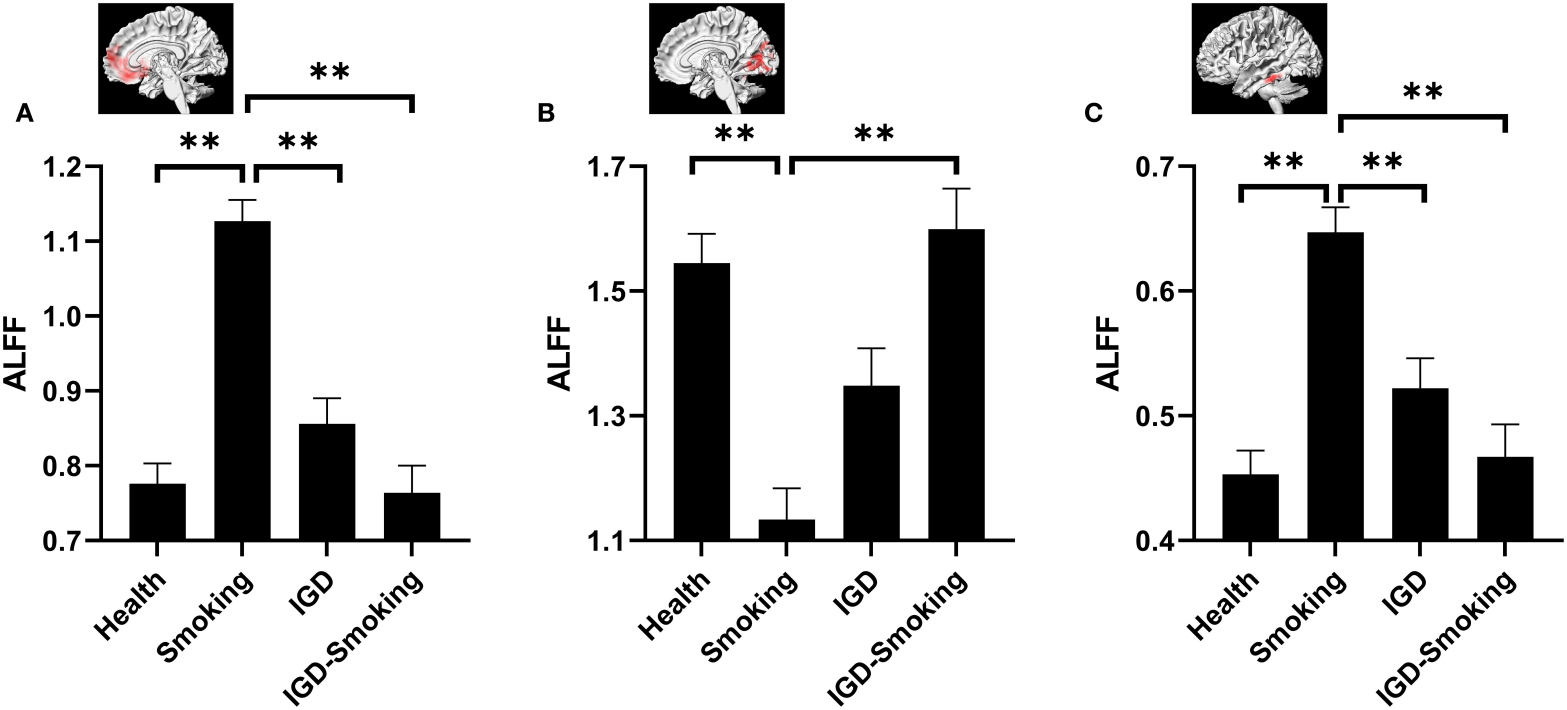
*Post-hoc* analyses of ALFF values among the four groups. **(A)** The brain regions involved in the right MPFC/ventral striatum. ALFF in smoking group was significantly higher than healthy group (*p* = 3.61 × 10^−15^) while no significant ALFF differences between IGD-Smoking and IGD group were found (*p* = 0.439). There were no significant ALFF differences between IGD group and healthy group (*p* = 0.441) while IGD-Smoking group showed significantly lower ALFF than smoking group (*p* = 1.16 × 10^−12^). Moreover, ALFF in smoking group exhibited significantly higher value than IGD group (*p* = 5.55 × 10^−8^). **(B)** The brain regions involved the bilateral cerebellar and visual-related regions. ALFF in smoking group was significantly lower than healthy group (*p* = 1.17 × 10^−7^) while the difference between IGD–Smoking and IGD group did not survive the Bonferroni correction at *p* < 0.0083 (*p* = 0.038). No significant ALFF differences between IGD and healthy group was found (*p* = 0.081) while IGD-Smoking group showed significantly higher ALFF than smoking group (*p* = 2.32 × 10^−7^). **(C)** The brain regions involved the left temporal gyrus. ALFF in smoking group was significantly higher than healthy group (*p* = 8.55×10^−10^) while no significant ALFF differences between IGD-Smoking group and IGD group were found (*p* = 0.855). There were no significant ALFF differences between IGD group and healthy group (*p* = 0.20) while IGD-Smoking group showed significantly lower ALFF than smoking group (*p* = 1.00 × 10^−6^). Moreover, ALFF in smoking group exhibited significantly higher value than IGD group (*p* = 0.001). The graphs above the column group were the involved brain regions. **p < 0.05/6 = 0.0083 (Bonferroni correction).

The *post-hoc* pairwise comparison controlling for age, gender, educational level, and head motion (Bonferroni correction, *p* < 0.0083) demonstrated that ALFF showed no statistical differences between IGD group and healthy group (IGD group: 0.86 ± 0.03; Healthy group: 0.78 ± 0.03; *p* = 0.441) while IGD-Smoking group showed significantly lower ALFF when compared to smoking group (IGD-Smoking group: 0.76 ± 0.04; Smoking group: 1.13 ± 0.03; *p* = 1.16 × 10^−12^) in the right MPFC/ventral striatum (Figure 2A). In the bilateral cerebellar and visual-related regions, IGD group showed no statistical differences of ALFF with healthy group (IGD group: 1.35 ± 0.06; Healthy group: 1.55 ± 0.05; *p* = 0.081) while IGD-Smoking group showed significantly higher ALFF when compared to smoking group (IGD-Smoking group: 1.60 ± 0.07; Smoking group: 1.13 ± 0.05; *p* = 2.32 × 10^−7^, Figure 2B). As for the left temporal gyrus, IGD group showed no statistical differences of ALFF with healthy group (IGD group: 0.52 ± 0.02; Healthy group: 0.45 ± 0.02; *p* = 0.20) while IGD-Smoking group showed significantly lower ALFF when compared to smoking group (IGD-Smoking group: 0.47 ± 0.03; Smoking group: 0.65 ± 0.02; *p* = 1.00 × 10^−6^, Figure 2C).

### Effect of Smoking on Healthy and IGD Groups

The *post-hoc* pairwise comparison controlling for age, gender, educational level, and head motion (Bonferroni correction, *p* < 0.0083) also showed that ALFF in smoking group was significantly higher than healthy group in the right MPFC/ventral striatum (*p* = 3.61 × 10^−15^) and the left temporal gyrus (*p* = 8.55 × 10^−10^) while IGD-Smoking group showed no statistical differences of ALFF when compared to IGD group (*p-*value was 0.439 and 0.855, respectively) (Figures 2A,C). In the bilateral cerebellar and visual-related regions, smoking group showed significantly lower ALFF than healthy group (*p* = 1.17 × 10^−7^). Moreover, the ALFF difference between IGD-Smoking and IGD group (*p* = 0.038) did not survive the Bonferroni correction at *p* < 0.0083 (Figure 2B).

In addition, compared to the healthy control group, smoking caused significantly larger ALFF changes than IGD in the right MPFC/ventral striatum (*p* = 5.55 × 10^−8^, Figure 2A), as well as the left temporal gyrus (*p* = 0.001, Figure 2C). Although the difference between IGD and smoking group did not survive the Bonferroni correction at *p* < 0.0083, it was approaching significance (*p* = 0.047) in the bilateral cerebellar and visual-related regions (Figure 2B). However, ALFF in IGD-Smoking group was not significantly different with healthy group in these regions (*p* = 1.00).

### Correlations Between ALFF and Clinical Characteristics

**Figure 3:**
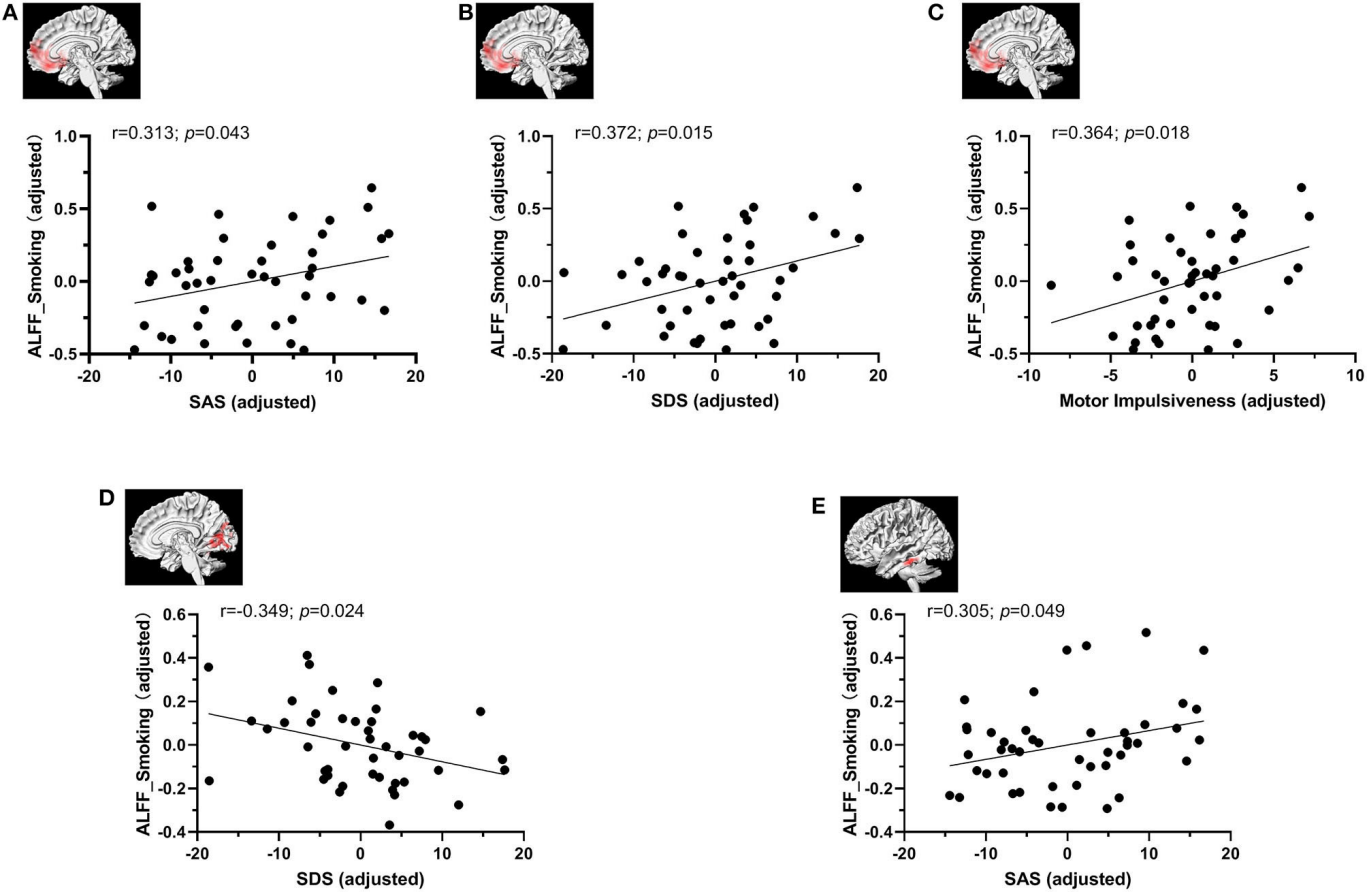
The correlation analysis between ALFF values and clinical characteristics controlling for age, gender, educational level, and head motion. The ALFF value in the smoking group was associated positively with SAS **(A)**, SDS **(B)** and motor impulsiveness dimension of BIS-11 scores **(C)** in the right MPFC/ventral striatum. **(D)** The ALFF value in the smoking group had a trend toward negative correlation with SDS score in the bilateral cerebellar and visual-related regions of lingual and calcarine gyrus and cuneus. **(E)**The ALFF value in the smoking group was associated positively with SAS score in the left temporal gyrus.

The ALFF values from significant clusters showing interaction effects were extracted to perform correlation analysis with clinical characteristics controlling for age, gender, educational level and head motion (Figure 3). We found that the participants in smoking group with higher ALFF values exhibited higher SAS (*r* = 0.313; *p* = 0.043), SDS (*r* = 0.372; *p* = 0.015) and motor impulsiveness dimension of BIS-11 (*r* = 0.364; *p* = 0.018) scores in the right MPFC/ventral striatum and SDS (*r* = −0.349; *p* = 0.024) scores in the bilateral cerebellar and visual-related regions, and higher SAS score (*p* = 0.049, *r* = 0.305) the left temporal gyrus. ALFF of other three groups did not show significant correlation with smoking related variables (i.e., duration of smoking, age at first smoking, and FTND), IGD related variables (CIAS) and questionnaire scores of SAS, SDS, BIS, and its three dimensions.

## Discussion

As far as we know, this is the first study to explore interaction between smoking and IGD on spontaneous brain activity using rs-fMRI. Our findings demonstrated that there were significant smoking by IGD interactions on ALFF in the right MPFC/ventral striatum, left temporal gyrus, and bilateral cerebellar and visual-related regions. Specifically, in the right MPFC/ventral striatum and left temporal gyrus, ALFF in smoking group was significantly higher than healthy group while there were no significant ALFF differences between IGD-Smoking group and IGD group. There were no significant ALFF differences between IGD group and healthy group while IGD-Smoking group showed significantly lower ALFF than smoking group. However, in the bilateral cerebellar and visual-related regions, ALFF in smoking group was significantly lower than healthy group while ALFF in IGD-Smoking group did not show significant difference with IGD group. There were no significant ALFF differences between IGD group and healthy group while IGD-Smoking group showed significantly higher ALFF than smoking group. Taken together, these findings suggest that smoking and IGD interact with each other while they work in human brain, especially in reward and motivation functions related regions.

Smoking group demonstrated significantly higher SAS, SDS, and impulsivity scores, which is consistent with previous studies that smoking was associated with anxiety and depression with smoking (34). Anxiety was one of the strongest predictors of nicotine intake and nicotine-seeking behavior (35) and significantly related to the severity of cigarette dependence and unsuccessful attempts to quit (36). Similar reports have been made of depression that compared with persons without depression, depressed individuals are more likely to smoke and relapse, less likely to quit (37). In turn, smoking appears to increase the risk for the development of depression (38). On the side of initiation and of the persistence of internet addiction, depression was also an important predicting factor (9) and it increased linearly with YDQ score (39). In addition, there were significant correlations between internet addiction with depression and anxiety (40). Specific internet addicts showed higher social anxiety (41). In addition, impulsivity components differentially predicted tobacco use (42) and higher impulsivity determined by the BIS increased adolescents' odds of being smokers (43) and smokers reported higher impulsivity on the BIS-11 than never smokers (44). No significant correlation between SAS and SDS with IGD or IGD-Smoking in this study might be due to the small sample size.

The finding that in the right MPFC/ventral striatum and left temporal gyrus, smoking group had significantly higher ALFF than healthy group is consistent with the observation of increased activation in the ACC and superior temporal gyrus in the nicotine group compared with the controls (45). It suggested that compared to healthy group, these brain regions were more vulnerable to smoking. However, no significant ALFF differences between IGD-Smoking group and IGD group in these brain areas were found. In other words, when both IGD and smoking co-existed, ALFF of these brain regions were identical to that of the healthy control group. These two results demonstrated that when smoking occurred in the healthy and IGD group, the changes of the spontaneous brain activity were different between these two groups. It was reported that the youthful initiation of internet addiction in teenage students could be predicted by smoking, and substance use like smoking is likely to exacerbate internet addiction initiation and persistence (9). In addition, Sung et al. (24) proposed that smoking may associate with high risk of internet addiction. More interestingly, compared to schizophrenia non-smokers, schizophrenia smokers demonstrated reversed intrinsic brain activity in the pre-frontal cortex. In short, smoking had different effects on healthy and IGD group.

Additionally, in the same brain regions, we found no significant ALFF differences between IGD group and healthy group. Although many studies had found that IGD group had significant differences with healthy controls on the spontaneous brain activity in the right orbital frontal gyrus (46), the right ACC (19), and the temporal gyrus (47). The inconsistency with our finding may arise from the sample characteristics or correction method. For example, in this study, we using FWE correction in the two-way ANCOVA to find significant clusters and Bonferroni correction in the *post-hoc* pairwise comparison while the correction method was Alphasim correction in the report of Lin et al. (47). The study also revealed that IGD-Smoking group showed significantly lower ALFF than smoking group. Sung et al. (24) also suggested that compared with low-risk group for internet addiction, smoking rates were significantly higher among high-risk group for internet addiction. This cannot be explained that IGD causes smoking. It might be that they have the similar causal factors. This may make the phenomenon that IGD individuals in the internet cafes often tended to be smokers and the ALFF of IGD—Smoking group did not show significant difference with healthy controls better understood. To sum up, these results in this study illustrated that when healthy and smoking group were addicted to IGD, the changes of the spontaneous brain activity were different between these two groups.

In this study, there was significant interaction between IGD and smoking in the right MPFC/ventral striatum and left temporal gyrus. The MPFC is considered as promoting goal-directed behavior by assessing the incentive-significant stimuli and choosing to generating the desired results (48–50). It integrates the body signals to help make decisions which involved in creating and maintaining the expectations of rewards associated with reinforcement (51). The activity of medial OFC and ACC was highly correlated with the subjective valuation of drug-related stimuli (52) and showed increased activity after individuals receiving a reward or before an expected reward immediately (53). The glucose metabolic activity of OFC in IGD adolescents also showed abnormal increase when compared with controls. (54). So the brain activity in the frontal gyrus might be perceived as a marker to reflect the reduced cognition and control ability of addiction (15) and enhanced reward sensitivity (46). Increased functional connectivity of OFC was also observed in cannabis users (55). However, in heroin-dependent individuals, regional homogeneity was diminished in the medial OFC (22). In methamphetamine users, metabolic activity of OFC was reduced (56). The medial OFC is a part of the limbic system, which is involved in decision making and expectation (57). In these studies, the OFC demonstrated conflicting results, which, on the one hand, may be due to the small sample size and different addictive substance with different mechanism. On the other hand, ALFF measures the average value over a period of time, which could not reflect the dynamic property of real brain signal. Further dynamic investigation will be developed to obtain more evidence to understand the potential neural mechanism behind addiction disorder.

Activation was also found in ACC in IGD group when compared to the control group in an event-related fMRI study (58). Another fMRI study found that global cerebral blood flow in IGD subjects appeared to be significantly higher in ACC which may related to attention and arousal mediation (59). ACC involves in making the information of emotion and motivation, regulation of reward-seeking behavior as well as attention salient (60, 61) and processing memory and encoding the motivation of substance cue (62, 63). It is crucial in monitoring the meaning of the stimulus relative to the target. Once the appetite cues are identified, ACC will determine whether and how strong the behavioral response is (64, 65). As for other kinds of addiction disorders, for instance, in alcohol use disorders activated the left cingulate gyrus and left superior temporal gyrus was founded (62). Significant activation of ACC was also observed in cocaine abusers (66). However, Volkow et al. found decreased metabolism in cingulate gyrus and OFC in cocaine abusers. Cocaine abusers also showed significant decreases in DA release which leads to decreased activation of reward circuits and further leads to compulsive drug administration to perpetuate cocaine use as a means to compensate for this deficit (67). Several studies demonstrated that the ACC is associated with selective attention (68, 69) which is a prerequisite for signaling reward (70). As a part of a corticostriatal circuit, the ACC plays an important role in stimulus–reward learning (71, 72). Once appetitive (and aversive) cues are identified by the visual cortex and motivational/attentional centers, ACC are activated to reinforce the processing (73). The ACC activation was also observed during cognitive processes which could be dysfunctional after anterior cingulotomy in human (74).

Increased fractional ALFF (fALFF) was found in the superior temporal gyrus and the volume of temporal lobe were significantly reduced in addiction persons (19). The primary function of the temporal lobe is regulating sensory perception including processing vision and auditory. The activated inferior temporal gyrus might serves as positive intensifying factor to reveal oneself contacting addiction behavior repetitively (19). What's more, the ALFF value in the smoking group was associated positively with SAS and SDS scores in these brain regions, indicating the more anxious, depressed, the higher ALFF in the smoking group. At the same while, the ALFF value in the smoking group was associated positively with SAS score in the left temporal gyrus. Given the function of these brain regions, we hypothesized that smoking might be due to anxiety, depression. Additionally, it also illustrates that ALFF can be regarded as a reliable marker in the exploration of the brain function.

In the bilateral cerebellar and visual-related regions of lingual and calcarine gyrus and cuneus, we found that ALFF in smoking group was significantly lower than healthy group. Activity in the bilateral cuneus and lingual gyrus was also found diminished in heroin-dependent individuals (22). However, ALFF in IGD–Smoking group did not show significant difference with IGD group. These two findings demonstrated that when smoking had different effects of spontaneous brain activity on healthy and IGD group. In addition, this study also illustrated no significant ALFF differences between healthy group and IGD group while IGD–Smoking group showed significantly higher ALFF than smoking group. All these results pointed out the interaction between IGD and smoking. A functional MRI study supported that the cerebellum serves as cognitive functions (75). Researchers have found a correlation between structural abnormalities in the cerebellum and the clinical manifestations of certain psychiatric disorders. The cerebellum has lots of functional connection with the brain, which, to some extent, contributes to regulating the cognition, emotions and thinking. There are also reports which found the cerebellar structural abnormalities is correlated with certain mental illness (76). The decreased activation of cerebellum of our study might suggest the impairment of cerebellum in IGD individuals and smokers with abnormality of cognitive functions. The cuneus involves in visual processing inhibitory control centers. The regional homogeneity of heroin-dependent individuals was found diminished in the bilateral cuneus (22). Moreover, the ALFF value in the smoking group had a trend toward negative correlation with SDS score in the bilateral cerebellar and visual-related regions of lingual and calcarine gyrus and cuneus, suggesting that smokers with lower ALFF values in these brain regions felt more depressed.

Whether smoking caused significant ALFF changes in healthy group but not in IGD group, or IGD caused significant ALFF changes in smoking group but not in healthy group, it demonstrated the interaction of the two factors and the different combined effect from the single effect of one factor. IGD was related to smoking (40). More interestingly, nicotine addiction effect independent of other addictions like gambling (77) and disease like schizophrenia (78) and IGD associated with harmful alcohol use among college students was also found (17, 79). Compulsive internet use might have a causal relationship with changes in substance use in female (80). Yen proposed that the comorbidity of internet addiction and other substance addiction might indicate the predictive relationship between them (81). IGD and smoking might share the similar neurobiological mechanisms (8, 10). IGD and smoking is the typical representative of behavioral and substance addiction, respectively. The underlying mechanism about their different performance in different populations are still unknown, so further researches are need to determine.

Furthermore, in the significant brain areas of ANCOVA, we also found that compared to healthy controls, the increased ALFF in smoking group was significantly larger than IGD group in the right MPFC/ventral striatum. It illustrated that healthy controls might be more sensitive to smoking than IGD. The reason might be that in smokers, substance like nicotine or other aspects in cigarette worked while there wasn't any substance in IGD individuals.

This study has several limitations. First, the possible influences of alcohol cannot be excluded, as alcohol consumption was not quantitatively evaluated. Although most subjects self-reported no or seldom daily alcohol consumption, whether the subjects deceptively reported of alcohol dependence could not confirmed. Therefore, the possible effects of alcohol use on the interactions between smoking and IGD need to be studied in the future. Second, variables of age, sex and education level were not well-matched among the four groups although they went to the statistical procedure as controlling factors. Several studies showed sex-specific effects of cigarette smoking on rsFC (82) and IGD on ALFF and rsFC (83). Child abuse increases risk for substance use in part (84) and there were significantly differences in light and heavy smokers (20). Although they served as covariates, we cannot completely exclude the potential impacts of these factors. Third, no significant correlation remained after Bonferroni correction. This might be related to the small sample size and future research of large sample size is needed to help us to get deep understanding of the relationship between common scales of psychological disorder and IGD and Smoking. Finally, although this resting-state fMRI study investigated interaction between smoking and IGD on spontaneous brain activity, future task dependent examination will help us to have a better understanding of the neural mechanism of IGD and smoking.

In conclusion, we demonstrated that smoking and IGD were not independent and they actually interacted with each other on spontaneous brain activity, mainly characterized by significant interaction in the right MPFC (i.e., orbital frontal gyrus, anterior cingulate cortex and medial frontal gyrus) extending to the right ventral striatum, bilateral cerebellar and visual-related regions (i.e., lingual and calcarine gyrus and cuneus) as well as the left temporal gyrus. Our results may imply the fact that IGD people are more tended to get smoking addiction. It may also be possible to predict that smoking addiction person may be more easily to get internet addiction than healthy people. Our findings may have the possibility to improve our understanding of the latent neurological theory and mechanism by which several addictive factors work together on human brain.

## Data Availability Statement

The raw data supporting the conclusions of this article will be made available by the authors, without undue reservation.

## Ethics Statement

The studies involving human participants were reviewed and approved by the Medical Ethics Review Board of Renji Hospital, Shanghai Jiaotong University. Written informed consent to participate in this study was provided by the participants' legal guardian/next of kin.

## Author Contributions

FL and HL contributed to the study design. XH, YW, WD, YS and YZ collected the fMRI data. FL and XQ analyzed the MRI data and drafted the manuscript. All authors contributed to the article and approved the submitted version.

## Conflict of Interest

The authors declare that the research was conducted in the absence of any commercial or financial relationships that could be construed as a potential conflict of interest. The reviewer, T-FY, declared a shared affiliation, though no collaboration, with several of the authors, XH, YW, WD, YS, and YZ, to the handling Editor.
